# Incidence and risk factors for ventilator associated events in uganda´s general ICUS

**DOI:** 10.1186/2197-425X-3-S1-A381

**Published:** 2015-10-01

**Authors:** C Namutebi, A Kwizera

**Affiliations:** Anaesthesia, Makerere University, Kampala, Uganda; Makerere University, Kampala, Uganda

## Introduction

Mechanical ventilation is the ultimate form of respiratory support commonly given to the critically ill in the ICU. It is commonly complicated by Ventilator Associated Events (VAEs) which increase mortality and length of stay in ICU among ventilated patients. Various studies have been carried out to determine the incidence and associated factors but none in Uganda.

## Objectives

To determine the incidence and risk factors for ventilator associated events in Uganda's general intensive care units.

## Methods

No previous study had been carried out in Uganda. This study was a prospective cohort study with a total of 97 mechanically ventilated adults enrolled but 83 were analysed for the incidence and risk factors for VAEs in Uganda's general ICUs between February 2014 and January 2015. Data was entered with EpiData 3:1 and analysed with STATA version 12.

## Results

We found 58/83 (69.9%) patients developed VAEs. Of these, 32/83 (39.0%) had VAC, 27/83 (32.5%) had infection related ventilator- associated conditions (IVACs) and 31/83 (38.3%) had probable VAP.

At multivariate analysis, a high diastolic blood pressure (OR 0.78; 95% CI 0.64-0.95, p = 0.014), high GCS (OR 0.37; 95% CI 0.14-0.98, p = 0.045) and longer duration of ventilation (OR 0.32; 95% CI 0.13-0.78, p = 0.012) reduced the odds of development of VAC while SIMV mode of mechanical ventilation (OR 4.63; 95% CI 1.04-20.63) increased the odds of development of VAC. Female patients at multivariate analysis (OR 0.31; 95% CI 0.01-0.99, p = 0.047) had a reduced association in development possible VAP compared to their male counterparts. No significant association was found at multivariate analysis for risk factors for IVAC and probable VAP.

Known risk factors including blood transfusion, COPD, age > 60 years, head trauma, ARDS, antibiotics given < or > 48 hours of admission, abdominal surgery within 24 hours of admission, transportation out of ICU, reintubation, supine position, enteral feeding and paralysis were not associated with VAEs including the VAP prevention bundle which is proven to be protective with chlorhexdine mouth wash, semirecumbent positioning, DVT prophylaxis, stress ulcer prophylaxis with PPIs or H2 receptor blockers, daily spontaneous breathing trials, and sedative interruptions.

The most frequently isolated organisms were *Acinetobacter species* (37.5%)*, K. pneumonia* (35.3%), *Pseudomonas auroginosa* (14.7%), *E. coli* (11.7%), *S. aureus* (5.9%) and *Enterobacter species* (5.9%) sensitive to commonly Amikacin, Imipenem and PISA.

## Conclusions

VAEs are more common and morbid. VAE prevention bundles are needed in our setting.

